# Spin Rate Effects in a Micromachined Electrostatically Suspended Gyroscope

**DOI:** 10.3390/s18113901

**Published:** 2018-11-12

**Authors:** Boqian Sun, Shunyue Wang, Yidong Tan, Yunfeng Liu, Fengtian Han

**Affiliations:** Department of Precision Instrument, Tsinghua University, Beijing 100084, China; sunboqian@mail.tsinghua.edu.cn (B.S.); wsy15@mails.tsinghua.edu.cn (S.W.); tanyd@mail.tsinghua.edu.cn (Y.T.)

**Keywords:** MEMS, micromachined spinning-rotor gyroscope, electrostatic suspension, spin rate, scale factor, noise, resolution, bias instability

## Abstract

Spin rate of a high-speed spinning-rotor gyroscope will make a significant impact on angular rate sensor performances such as the scale factor, resolution, measurement range, and bias stability. This paper presents the spin rate effects on performance indicators of a microelectromechanical systems (MEMS) gyroscope where a free-spinning rotor is electrostatically suspended in an evacuated vacuum cavity and functions as a dual-axis angular rate sensor. Theoretical models of the scale factor and measurement range of such a spinning-rotor gyroscope are derived. The experimental results indicate that the measured scale factors at different settings of the spin rate match well with the theoretical predication. In order to separate the disturbance component of the rotation control loop on the gyroscope output, a testing strategy is proposed by operating the gyroscope at different spin rates. Experimental results on a prototype gyroscope show that the squared drive voltage generated by the rotation control loop is approximately proportional to the noise of the gyroscope output. It was further investigated that an improved performance of such spinning-rotor gyroscopes can be achieved by operating the gyroscope rotor at an optimal spin rate.

## 1. Introduction

High-speed spinning-rotor gyroscopes with decades of the accumulated knowledge and experiences on conventional mechanical gyroscope technologies [1], such as dynamically tuned gyros [2], liquid-floated gyros, and electrostatically suspended gyros, have been used widely in tactical and navigation grade applications so far [3,4,5]. Benefitting from the micro-fabrication technology emerging in the 1990s, many new miniaturized gyroscopes have come out and aim at reducing volume and power consumption, lowering cost, and maintaining precision as much as possible [6].

For the sake of isolating the spinning rotor from unexpected mechanical friction, various micro-machined spinning-rotor gyroscopes with different suspension schemes including liquid suspension [7], gas-lubricated suspension [8], electromagnetic bearing, and electrostatic bearing have been successfully fabricated in order to achieve high accuracy [9,10,11]. The micromachined electrostatically suspended gyroscope (MESG), which is more compatible with the microelectromechanical systems (MEMS) fabrication process and easily achieves a high spin rate, has a potential to realize a high performance spinning-rotor gyro [12,13,14].

The rotor of a MESG is usually electrostatically suspended in vacuum by a contactless bearing in five degrees of freedom and spins up to over 10^4^ rpm (revolutions per minute). Currently, MESG has demonstrated its potential tactical-grade performance [15]. Several MESG prototypes were introduced where most of the published research focused on the structure design [16,17], fabrication [16], electrostatic suspension [15,17,18], rebalance loop control [19], experimental performance test [20], error analysis, and mechanical thermal noise [21,22].

In general, the overall performance of the spinning-rotor gyroscopes depends on multiple design parameters and suppression on various error sources including micro-fabrication tolerances, imperfect control electronics and electrical noises, stress deformation of the sensing structure, inherent temperature drift, and electromagnetic disturbances from the surrounding environment [19]. Therein lies a significant factor, that is, the spin rate of the rotor, which directly determines the measurement range and scale factor of such spinning-rotor gyroscopes. Heretofore, little research has been conducted on the gyroscope performance optimization by considering the spin rate setting and associated rotation control effects. This paper will focus on theoretical analysis of the spin rate effects on the MESG performance and experimental evaluation in our updated MESG prototype.

## 2. Description of Micromachined Electrostatically Suspended Gyroscope

### 2.1. Gyroscope Structure

**Figure 1:**
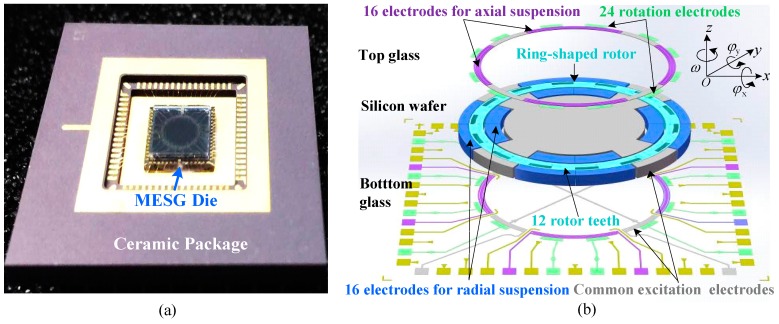
Micromachined electrostatically suspended gyroscope (MESG), (**a**) a ceramic packaged device and (**b**) the exploded view of rotor and electrode configuration.

A fabrication process for the MESG device based on glass/silicon/glass triple wafer bonding and bulk micromachining has been developed successfully. Figure 1a illustrates a fabricated MESG device with a ceramic package. The MESG sensing structure comprises 16 common excitation electrodes, 24 rotation electrodes, 16 axial and 16 radial suspension electrodes, and a ring-shaped rotor. The contactless rotor is electrostatically suspended in five degrees by axial and radial suspension electrodes jointly and is driven by rotation electrodes functioned as a three-phase variable-capacitance electrostatic motor [23]. The rotor and radial suspension electrodes are etched and formed on the middle conductive silicon wafer, while the axial electrodes and rotation electrodes symmetrically located above and underneath the rotor, respectively, are sputtered and patterned on the top/bottom glass wafers, as shown in Figure 1b. The common electrodes are distributed evenly on the silicon wafer and top/bottom glass wafers, which are electrically connected together and used to apply high-frequency excitation for five degree of freedom (DOF) capacitive position sensing.

The ring-shaped rotor, with an outer radius of 2.0 mm, an inner radius of 1.73 mm, and a thickness of 68 μm, has to be operated in vacuum so as to greatly reduce the viscous air–film damping effect and achieve a high spin rate. Both suspension and high-speed rotation of the free rotor by controlled electrostatic filed make such a structure design act as one kind of miniaturized free spinning-rotor gyroscopes [20]. As depicted in Figure 1b, the spin rate is ω and the two angular-rate sensing axes are $\varphi_{x},\varphi_{y}$.

### 2.2. Operating Principle of MESG

Functioning as a spinning-rotor gyroscope, the angular motion of the moving vehicle relative to the inertial space can be detected by measuring the variation of the angular position or angular velocity of the gyroscopic case around the two sensing axes that are orthogonal to the spin axis, and provided by the outputs of spinning-rotor gyroscopes.

**Figure 2:**
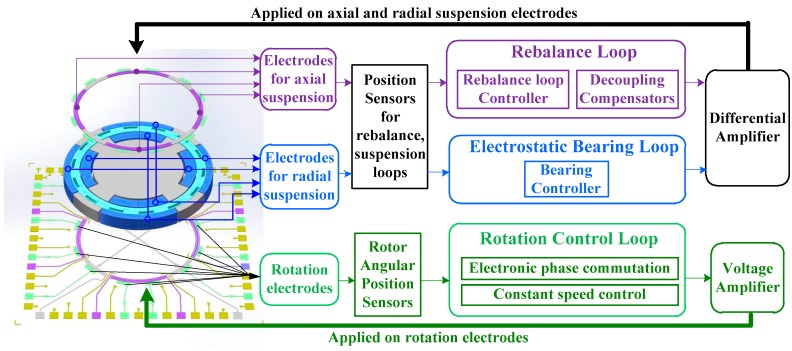
Block diagram of the control loop including rebalance loop, electrostatic bearing, and rotation control.

A combination of electrostatic bearing, rotation control, and rebalance loop constitutes the whole control system for a MESG, as depicted in Figure 2 [19]. Firstly, the electrostatic bearing loop supports the rotor at the geometric center of axial and radial suspension electrode cavity in five DOFs. The motion of the rotor is acquired via position sensors and regulated by the controlled electrostatic force that is updated by an electrostatic bearing controller. Secondly, a rotational control loop—to be exact, an electronic commutation scheme with spin-up and constant-speed operation—is able to rapidly accelerate the free-spinning rotor in step and maintain constant spin rate at high precision despite external disturbances, in order to maintain angular momentum conservation of the spinning rotor. Finally, a rebalance loop benefitting from the previous two loops is able to output the measured dual-axis angular rate signals while any input angular motion along the sensing axes occurs. Note that all drive electrodes are designed as differential pairs so as to achieve virtual ground on the spinning rotor.

### 2.3. Scale Factor and Measurement Range of MESG

Scale factor and measurement range are two main parameters in the design of MESGs. Spinning-rotor gyroscopes are able to maintain a relatively large angular momentum while the rotor is spinning at high speed. Under an input angular rate $\Omega_{x}$, the rebalance loop of such gyroscopes will output the gyroscope signal (voltage or current) reflecting the real-time value of $\Omega_{x}$, and can be derived as Equation (1).

(1) $$\left|H\Omega_{x}\right|=V_{\Omega r}K_{\mathrm{vr}}\;$$

where $V_{\Omega r}$ describes the output voltage of the MESG; $H=J_{z}\omega_{}$ is the angular momentum of the rotor and $J_{z}=\pi \rho h((r_{o}^{4}-r_{i}^{4})/4$ represents the rotor moments of inertia about *z*-axes; ρ is the density of silicon rotor; $r_{o},r_{i}$, and h denote the outer, inner radius, and thickness of the rotor, respectively. $K_{\mathrm{vr}}$ is the torquer gain of the rebalance loop that can be calculated by $K_{\mathrm{vr}}=8\varepsilon V_{\mathrm{pr}}(r_{\mathrm{zo}}^{3}-r_{\mathrm{zi}}^{3})sin(\alpha /2)/3d_{z}^{2}$, where $V_{\mathrm{pr}}$ stands for the constant bias voltage; $d_{z}$ is the axial gap; $r_{\mathrm{zo}},r_{\mathrm{zi}}$, and α represent the outer, inner radius, and arc angle of the axial suspension electrodes, respectively; ε is the dielectric constant in vacuum. Then, the ideal scale factor $K_{f}$ (in V/(°s^−1^)) can be modeled by substituting $\Omega_{x}=1{}^{\circ}/s$ into Equation (1).

(2) $$K_{f}=\frac{\left|H\Omega_{x}\right|}{K_{\mathrm{vr}}}=\frac{3\omega_{}\pi^{2}\rho h(r_{o}^{4}-r_{i}^{4})d_{z}^{2}}{180\times 16\varepsilon V_{\mathrm{pr}}(r_{\mathrm{zo}}^{3}-r_{\mathrm{zi}}^{3})\sin(\alpha /2)}\;$$

The maximum measurement range of the MESG (in °/s) can be further obtained by the following:

(3) $$\Omega_{r\_\max}=\frac{180\times 16\varepsilon V_{\mathrm{pr}}{}^{2}(r_{\mathrm{zo}}^{3}-r_{\mathrm{zi}}^{3})\sin(\alpha /2)}{3\pi^{2}\rho h\omega_{}(r_{o}^{4}-r_{i}^{4})d_{z}^{2}}\;$$

### 2.4. Scale Factor Influenced by Spin Rate

Reformatting Equation (2) for an explicit expression, the scale factor of a spinning rotor gyroscope is proportional to the spin rate $\omega_{}$ in theory, as given by the following:

(4) $$K_{f}=\frac{3\pi^{2}\rho h(r_{o}^{4}-r_{i}^{4})d_{z}^{2}}{180\times 16\varepsilon V_{\mathrm{pr}}(r_{\mathrm{zo}}^{3}-r_{\mathrm{zi}}^{3})\sin(\alpha /2)}\omega_{},$$

then, $\Delta \omega_{}$ (in rad/s), the error of $\omega_{}$ such as the jitter and slowly-changing drift of the spin rate, will affect the repeatability and stability of $K_{f}$. Moreover, the accuracy of the gyroscope output will also suffer from the error of $K_{f}$, resulting in the performance degradation on the resolution and stability. According to Expression (4), the theoretical value of $K_{f}$ for MESG reaches 12.821 mV/(°s^−1^) at a rated spin rate 1 × 10^4^ rpm ($\omega_{}$ = $\omega_{0}$ = 1.0472 × 10^3^ rad/s). If $\omega_{}$ abnormally goes up to 1.0493 × 10^3^ rad/s, that is, 0.2 percent of the spin rate change resulting from the slowly-changing drift and jitter, in return, $K_{f}$ will be reduced to 12.795 mV/(°s^−1^). Therefore, the stability and repeatability of the scale factor are significantly affected by small changes of the spin rate. The lower the value of $\Delta \omega_{}$, the smaller the relative error of $K_{f}$ will be, making the spinning rotor gyroscopes accessible to a higher accuracy. With regard to a high resolution requirement of the MESG, the relative error of the spin rate deviation should be maintained below 10^−4^.

Expression (4) depicts the analytic form between the scale factor and spin rate, and to some extent, this means that the high spin rate will contribute to enhancing the sensitivity of the MESG. However, the micromachining fabrication tolerances including the flatness error of the rotor, alignment errors between the rotor, and electrode cavity will produce disturbing torque on the rebalance loop. Hence, the sensitivity of the gyroscope may be compromised with the increase of the rated spin rate, which will be discussed in Section 3.1.

### 2.5. Perturbed Torque of MESG

The MESG performance suffers inherently from some unmeasurable or inaccurate parameters so far, such as structural geometry error, residual air-film damping, and alignment error during glass-silicon anodic bonding. An effective way to separate one or two main error components from the output signal of the MESG should be addressed urgently. Five error sources of the output angular rate result from the electrostatic perturbed torque; rotation disturbance applied to the rebalance loop; inherent cross-axis coupling error in the dual-axis rebalance loop; discretization errors of the digital controller; and influence of the environmental conditions including temperature, air pressure, and magnetic disturbances. Among them, the electrostatic and rotational perturbed torque act as the dominant error components.

It is the electrostatic perturbed torque that is composed of a negative spring effect and an external acceleration associated with multiple error sources on geometrical structure including manufacturing tolerances of the suspension electrodes, axial mass unbalance and miscentering of the spinning rotor, mismatching of the rotor and electrodes assembly, and centrifugal deformation of the rotor spinning at high speed. The error factors of such nonlinear perturbed torque models become difficult to describe separately, which is expected to be evaluated via a multi-position drift error model test after miniaturization and vacuum packaging of the prototype MESG are completed [24].

The disturbance from the rotation loop to the rebalance loop is mainly composed of the spin rate fluctuation error and perturbed torque *M***_r_** generated by the rotation drive voltage [19]. Benefitting from the remarkable improvement of constant speed accuracy in previous research [20], it turns out that the perturbed torque from rotation drive voltages acts as a major source disturbing the rebalance loop. Assuming that an angular displacement $\theta_{y}$ exists, then an unexpected perturbed moment *M***_r_** will be generated and applied on the rotor. Based on the linearized equations of the gyro dynamics [19], such torque balance equation along one sensing axis can be derived as follows:

(5) $$\lambda \theta_{y}=M_{x}-M_{r}\;$$

where λ is the orthogonal-damping elastic coefficient, which is the product of the squeeze-film damping coefficient and rotor spin rate [19]; and *M***_x_** is the control moment corresponding to the bias angular position $\theta_{y}$. Considering the integral of moment elements over the whole rotation electrode area, the moment generated by the drive voltage and applied on the rotor can be calculated as Expression (6) with Taylor-expansion and linearization.

(6) $$\left\{ \begin{array}{l} M_{r}=k_{r1}V_{R}{}^{2}-k_{r2}V_{R}{}^{2}\theta_{y}\\ k_{r1}=\frac{12\varepsilon (R_{\mathrm{ro}}{}^{3}-R_{\mathrm{ri}}{}^{3})\sin(\alpha /2)}{3d_{z}{}^{2}}\\ k_{r2}=\frac{\varepsilon (R_{\mathrm{ro}}{}^{4}-R_{\mathrm{ri}}{}^{4})(\alpha +\sin\alpha )}{4d_{z}{}^{3}} \end{array}\right.\;$$

where *V***_R_** is the amplitude of the rotation drive voltage. $R_{\mathrm{ro}}$, $R_{\mathrm{ri}}$ and α represent the outer radius, inner radius, and arc angle of the rotation electrode, respectively. Finally, *M***_x_,** which perturbs the performance of a MESG, can be expressed as follows:

(7) $$M_{x}=(k_{r1}-k_{r2}\cdot \theta_{y})V_{R}{}^{2}+\lambda \theta_{y}\;$$

Equation (7) describes that *M***_x_** is jointly affected by the angular bias, damping, and rotation voltage. Obviously, it is the squared rotation voltage together with the angular bias that proportionally contributes to the perturbed moment *M***_x_**. In addition, the vacuum condition due to ambient temperature change and pressure regulation of the vacuum pump will also affect λ, so as to *M***_x_**. It is noted that $\lambda \theta_{y}$ produces a slowly-changing bias error in the gyroscope output. In order to figure out the dominate error items of such perturbed moment, a gyroscope noise experiment by operating the MESG at different spin rates is presented in Section 3.2.

## 3. Experiment Results and Discussion

**Figure 3:**
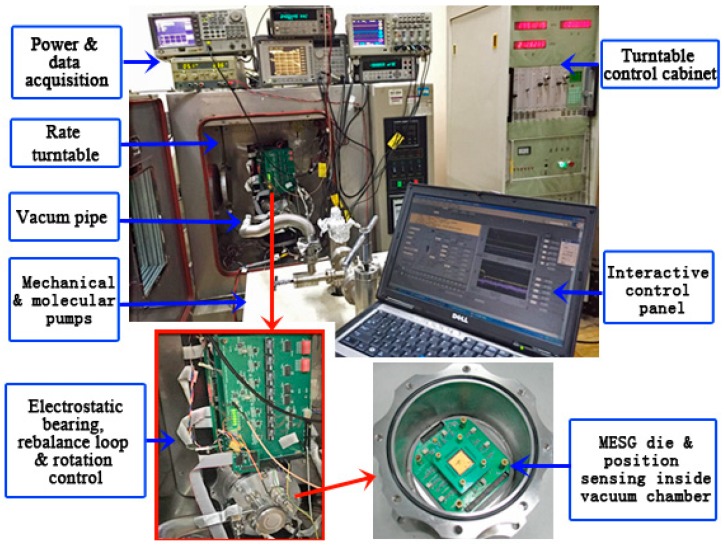
Test setup to study the spin rate effects on the MESG performance.

Figure 3 illustrates the test setup of the MESG mounted on a rate turntable. The MEMS die and associated position sensing electronics are fixed closely inside a small vacuum chamber for improving the signal-to-noise ratio of the weak position-sensing signals. The vacuum chamber and electrical circuits for the electrostatic bearing, rebalance loop, and rotation control are assembled on the turntable via a fixture. A turntable control cabinet is used to set the input angular rate at different settings. A vacuum pumping station consisting of both mechanical and turbo-molecular pumps is able to provide a stable vacuum in the vacuum chamber. A gauge outside the chamber is used to indicate current vacuum degree. Note that the air pressure inside the MESG die is not as low as the readout data of the gauge as a flow conductance exists between the die and gauge. A data acquisition system with a maximum sampling rate of 1 kHz is applied to record the gyroscope output while the MESG prototype can be operated at different settings of the spin rate. A virtual control panel realized on a host PC (Personal Computer) acts as the man–machine interface to operate MESG and record the data. Considering the spin rate effects on the gyroscopic performance, specifications such as the scale factor, resolution, and stability of a MESG prototype are experimentally evaluated in this section.

### 3.1. Scale Factor

Two methods exist to conduct the test of the gyroscope scale factor. First, the scale factor can be least-squares fitted via a series of input angular rate measurements provided by a rate turntable. Second, the scale factor is measured with the gyroscope output activated by the unit angular-rate input of the rate turntable. The nonlinearity, asymmetry, temperature drift, and repeatability of the scale factor are applied to evaluate overall input-output characteristics. Based on the experimental setup in Figure 3, the nonlinearity and asymmetry of the scale factor were tested.

**Figure 4:**
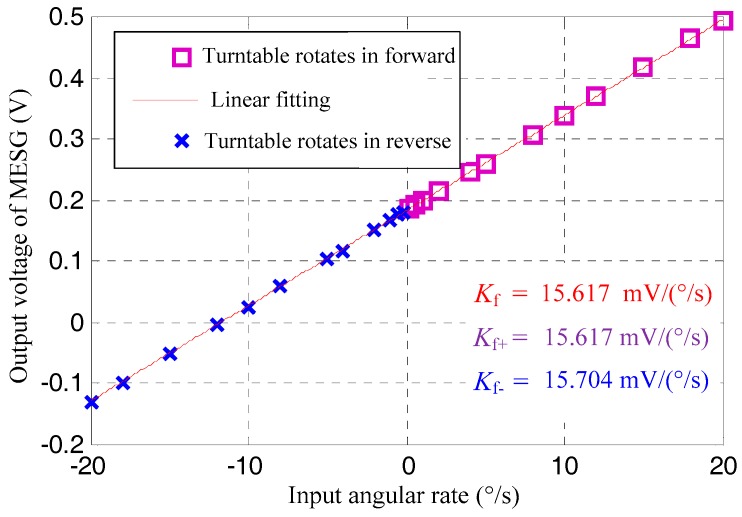
Scale factor test results.

While the MESG operated at 1.2 × 10^4^ rpm and was activated by the input angular rate provided by the turntable rotating in forward and reverse, the gyro output was recorded by the data acquisition system. The absolute values of the input angular rate increased step by step, including settings of ±0.2°/s, ±0.5°/s, ±1°/s, ±2°/s, ±4°/s, ±5°/s, ±8°/s, ±10°/s, ±12°/s, ±15°/s, ±18°/s, and ±20°/s. Every 30 sampling data of the gyroscope output was averaged at each input angular rate, and then the scale factor ($K_{f}$ = 15.61 mV/(°/s)) and its nonlinearity ($\nu_{f}$ = 0.361%) were calibrated by the linearity fitting using the method of least-squares, as shown in Figure 4. Next, by separating the gyro output data at turntable rotating in forward from that in reverse and least-squares fitting them likewise, $K_{f+}$ (turntable rotating in forward) and $K_{f}{}_{-}$ (turntable rotating in reverse) can be obtained. Then, the asymmetry of the scale factor, $\xi_{f}$, can be calculated by dividing the difference value of $K_{f+}$ and $K_{f-}$ by $K_{f}$.

For the MESG operating at 1.2 × 10^4^ rpm, the measured scale factor is 15.61 mV/(°/s), while its nonlinearity and asymmetry are 0.361% and 0.56%, respectively. Because the vacuum pipe is connected to the MESG chamber in order to maintain the high vacuum as represented in Figure 3, the testing range of the input angular rate is limited within 20°/s for a safe operation, which can be much increased after the MESG device is vacuum packaged in future work.

**Figure 5:**
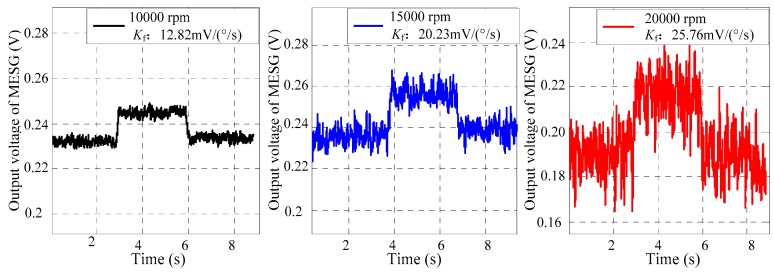
Scale factor tests of MESG at different settings of the spin rate.

On account of Expression (4) in Section 2.3, increasing the spin rate is an effective approach to increase the scale factor. Based on the MESG setup, the MESG was operated in the spin rate of 1 × 10^4^ rpm, 1.5 × 10^4^ rpm, and 2 × 10^4^ rpm driven by the rotation voltage of 4.2 V, 5.4 V, and 10.2V, respectively, at a vacuum pressure of 7.2 × 10^−3^ Pa read from the gauge. The single-axis rate turntable is able to provide the input angular rate of 1°/s along $\varphi_{x}$, and the data acquisition system can capture the output voltage of the MESG, contributing to analyzing the relation between the spin rate and scale factor. The experimental results in Figure 5 illustrate that the scale factor reaches 12.82 mV/(°/s), 20.23 mV/(°/s), and 25.76 mV/(°/s) at the rated spin rate of 1 × 10^4^ rpm, 1.5 × 10^4^ rpm, and 2 × 10^4^ rpm, respectively. However, it is noted that the output voltage noise increases gradually with the increased spin rate. Such rapidly-changing noise is mainly the result of the disturbing electrostatic torques generated by suspension and rotation control and applied on the miscentering rotor.

The measurement range can be confirmed commonly by the extreme angular rate testing via a rate turntable. However, the conventional extreme range test can not be performed on the MESG setup because of the vacuum pipe connecting the gyroscope with the pumps, which limits the allowable input angular rate. Therefore, the full-scale range was estimated by the measured scale factor with the formula $\Omega_{r\_\max}=V_{\mathrm{pr}}/K_{f}$, where the bias voltage of the MESG is 7.1 V. According to the measured results of the scale factor 12.82 mV/(°/s), 15.61 mV/(°/s), 20.23 mV/(°/s), and 25.76 mV/(°/s) at the spin rate of 1 × 10^4^ rpm, 1.2 × 10^4^ rpm, 1.5 × 10^4^ rpm, and 2 × 10^4^ rpm, respectively, the achievable measurement range is ±553.82°/s, ±454.63°/s, ±350.96°/s, and ±275.62°/s, respectively. In practice, the full-scale range should be less than the above estimation by a design margin for safe suspension of the rotor. Compared with the theoretical analysis of the scale factor and measurement range at different settings of the spin rate as listed in Table 1, the experimental results match the theoretical ones well with a relative error below 5.6%. This implies that the analytical model in Section 2.3 is helpful to guide the design of future MESGs.

### 3.2. Resolution and Noise

In this section, the static output signal of the MESG prototype is experimentally investigated in time and frequency domains, respectively.

**Figure 6:**
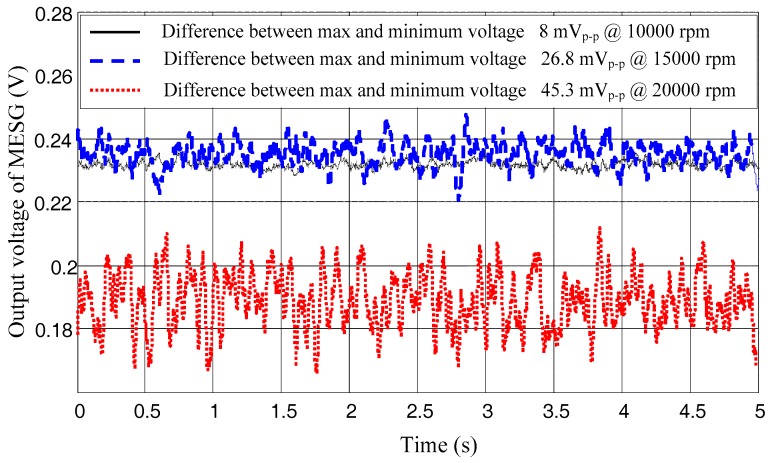
Static gyroscope outputs at different spin rates.

Driving the MESG at a constant spin rate of 1 × 10^4^ rpm, 1.5 × 10^4^ rpm, and 2 × 10^4^ rpm in a vacuum pressure of 9.1 × 10^−3^ Pa, 8.7 × 10^−3^ Pa, and 7.2 × 10^−3^ Pa, respectively, the gyroscope output in time domain is recorded by a digital multimeter with a sampling frequency of 1 kHz (Keysight 34410A), as depicted in Figure 6. Note that the actual vacuum inside the MESG die is estimated at around 10^−1^ Pa as a result of a large flow conductance between the MESG die and vacuum gauge. The peak-to-peak voltage noise is only 8 mV_p-p_ at 1 × 10^4^ rpm, yet it goes up to 45.3 mV_p-p_ at 2 × 10^4^ rpm. In addition, the bias voltages exist in the three curves owing to the effects of slowly-changing damping and angular position bias. Figure 6 shows that the frequency of the voltage noise is distributed up to around 5 Hz because of a low-pass filter used at the end-stage of gyroscope output with a bandwidth of 10Hz.

**Figure 7:**
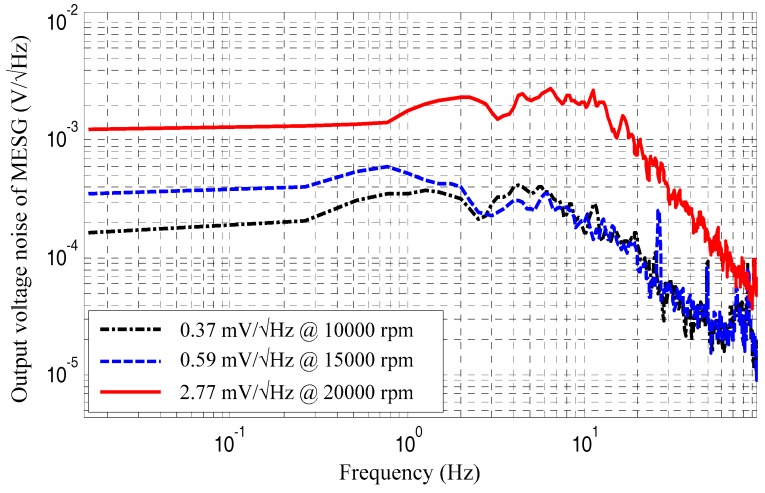
Spectrum analysis of static gyroscope output at different spin rates.

Power spectrum tests for the static output voltages were conducted via a dynamic signal analyzer (Agilent 35670A). The experimental results show that the noise spectrum increases 1.59 times from the gyro spin rate at 1.5 × 10^4^ rpm to 1 × 10^4^ rpm, and 7.48 times from 2 × 10^4^ rpm to 1.0 × 10^4^ rpm, as illustrated in Figure 7. As the rebalance loop will be disturbed by the moment of the rotation loop as mentioned in Section 2.5, the squared rotation drive voltage, angular bias, and damping work together to produce an unexpected perturbed moment on the spinning rotor. Although it is difficult to precisely obtain the real-time value of angular bias and damping coefficient during the MESG test, the rotation drive voltage is easy accurately to be measured. The squared rotation voltages and associated noise of the output voltages are listed in Table 2. The squared rotation voltage increases 5.89 times for the rotor spinning from 1 × 10^4^ rpm to 2 × 10^4^ rpm. Note that measured values at rows 5 and 6 in Table 2 are close and the discrepancy is less than 21.2%, which could be produced by the neglected disturbances including the angular bias and residual air damping. It is clear that the squared rotation voltages play as a dominant contribution to the noise of the MESG output voltage. It is estimated that a vacuum packaging of the MESG device with much lower air pressure below 10^−2^ Pa will decrease the required rotation drive voltage and thus greatly reduce the gyroscope output noise.

It has been demonstrated that the noise of the gyroscope output increases with a higher spin rate, which will further decrease the resolution of the MESG. Nevertheless, the higher spin rate contributes to enhancing the scale factor. A rate turntable test was conducted to compare the resolution at different spin rates. Based on an extension of previous research [20], the resolution measurements of the MESG are listed in Table 3. It is clear that the resolution improves from 0.018°/s at 1 × 10^4^ rpm to 0.012°/s at 1.5 × 10^4^ rpm, while it does not always increase even if the spin rate goes up to 2 × 10^4^ rpm. The resolution degradation may suffer from two factors in principle. One is the centrifugal deformation of the ring-shaped rotor spinning at high speed, and the other is the coupling error from the rotation control loop to the rebalance loop. Hence, a noise reduction of the gyroscope output will benefit from the optimization of the rotor geometry for higher rigidity and higher vacuum package for lower rotation drive voltage.

### 3.3. Bias Instability

Allan deviation acting as a method of the error analysis in time domain is widely applied to performance evaluation for inertial sensors. The bias instability is a major parameter to estimate the gyroscope performance, while the angular random walk reflects the long term drift of gyroscopes [15].

**Figure 8:**
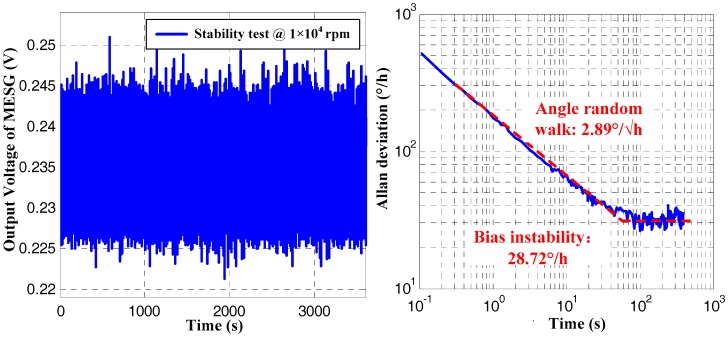
Gyroscope output in time domain and its Allan deviation at spin rate of 1.0 × 10^4^ rpm.

**Figure 9:**
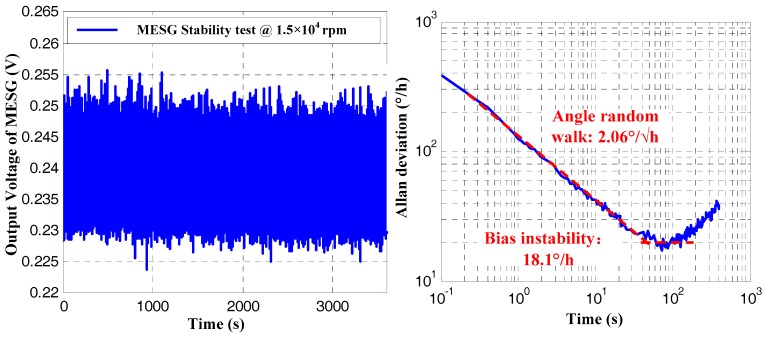
Gyroscope output in time domain and its Allan deviation at spin rate of 1.5 × 10^4^ rpm.

Operating the MESG prototype spinning constantly at 1 × 10^4^ rpm and 1.5 × 10^4^ rpm, the gyroscope output was sampled at 50 Hz for 2 h. Figure 8 shows the bias instability (BI) and angular random walk (ARW) are 28.72°/h and 2.89°/√h, respectively, at the rated spin rate of 1 × 10^4^ rpm. Further, when the spin rate increased to 1.5 × 10^4^ rpm, the BI and ARW are both improved and reach 18.1°/h and 2.067°/√h, respectively. It is noted that a drift error resulting from the weak vibration and temperature disturbance of the experimental environment occurs on the data segment after 300 s in the right side of Figure 9. Thus, the vacuum packaging of the MESG becomes a high priority to miniaturize the prototype, and improve both the pressure and temperature stability in our future research. Considering the above measured results comprising the scale factor, resolution, and bias instability, the spin rate plays an important role on the gyro performance. In this case, it is demonstrated that an optimal performance of the MESG prototype can be achieved by operating the gyroscope at about 1.5 × 10^4^ rpm.

## 4. Conclusions

The analytical and experimental results on performance improvements of a high-speed spinning-rotor gyroscope are described in this paper. The performance metrics such as the scale factor, resolution, and stability of a MESG prototype are studied theoretically and verified experimentally at different settings of the spin rate among 1 × 10^4^, 1.2 × 10^4^, 1.5 × 10^4^, and 2 × 10^4^ rpm, respectively. The experimental results indicate that the scale factor is in good agreement with the theoretical predication. The error analysis of such spinning-rotor gyroscopes indicates that the output voltage noise is approximately proportional to the squared rotation drive voltage. It is verified experimentally that an optimal performance of the MESG prototype can be achieved by setting the spin rate at 1.5 × 10^4^ rpm. In this case, the scale factor and resolution of the MESG are 20.23 m V/(°s^−1^) and 0.012°/s, the angular random walk and bias instability reach 2.067°/√h and 18.1°/h, respectively. The future research work will focus on miniaturizing the MESG setup and enhancing the vacuum stability of the MESG device via vacuum packaging.

## Figures and Tables

**Table 1:** Comparison between theoretical and experimental results at different spin rate settings.

Rated Spin Rate	1 × 10^4^ (rpm)		1.2 × 10^4^ (rpm)		1.5 × 10^4^ (rpm)		2 × 10^4^ (rpm)	
	Theory	Measured	Theory	Measured	Theory	Measured	Theory	Measured
Scale factor (mV/°/s)	12.8	12.82	15.4	15.61	19.2	20.34	25.6	25.75
Measurement range(°/s)	553.76	553.8	461.5	454.63	369.2	350.96	276.88	275.6

**Table 2:** Analysis of the micromachined electrostatically suspended gyroscope (MESG) output noises and rotation voltages at different spin rates.

Rated Spin Rate (rpm)	1 × 10^4^	1.5 × 10^4^	2 × 10^4^
P_0_ (Pa)	9.1 × 10^−3^	8.7 × 10^−3^	7.2 × 10^−3^
V_R_ (V)	4.2	5.4	10.2
V_R_^2^ (V^2^)	17.64	29.16	104.04
Voltage noise (mV/√Hz)	0.37	0.59	2.77
V_R_^2^/17.64 (1)	1	1.65	5.89
Voltage noise/0.37 (1)	1	1.59	7.48
(Row5 − Row6)/Row6	\	−3.7%	21.2%

**Table 3:** Measured resolution of the MESG at different spin rates [20].

Rated Spin Rate	1 × 10^4^ (rpm)	1.2 × 10^4^ (rpm)	1.5 × 10^4^ (rpm)	2 × 10^4^ (rpm)
Resolution (°/s)	0.018	0.014	0.012	0.012
